# Recent Advances in Sorbicillinoids from Fungi and Their Bioactivities (Covering 2016–2021)

**DOI:** 10.3390/jof8010062

**Published:** 2022-01-07

**Authors:** Xuwen Hou, Xuping Zhang, Mengyao Xue, Zhitong Zhao, Huizhen Zhang, Dan Xu, Daowan Lai, Ligang Zhou

**Affiliations:** State Key Laboratory of Agrobiotechnology, Department of Plant Pathology, College of Plant Protection, China Agricultural University, Beijing 100193, China; xwhou@cau.edu.cn (X.H.); zhangxuping5@cau.edu.cn (X.Z.); mengyaoxue@cau.edu.cn (M.X.); zhitongzhao@cau.edu.cn (Z.Z.); huizhenzhang@cau.edu.cn (H.Z.); cauxudan@cau.edu.cn (D.X.); dwlai@cau.edu.cn (D.L.)

**Keywords:** monomeric sorbicillinoids, bisorbicillinoids, trisorbicillinoids, hybrid sorbicillinoids, fungi, occurrence, biological activities

## Abstract

Sorbicillinoids are a family of hexaketide metabolites with a characteristic sorbyl side chain residue. Sixty-nine sorbicillinoids from fungi, newly identified from 2016 to 2021, are summarized in this review, including their structures and bioactivities. They are classified into monomeric, dimeric, trimeric, and hybrid sorbicillinoids according to their basic structural features, with the main groups comprising both monomeric and dimeric sorbicillinoids. Some of the identified sorbicillinoids have special structures such as ustilobisorbicillinol A, and sorbicillasins A and B. The majority of sorbicillinoids have been reported from fungi genera such as *Acremonium*, *Penicillium*, *Trichoderma*, and *Ustilaginoidea*, with some sorbicillinoids exhibiting cytotoxic, antimicrobial, anti-inflammatory, phytotoxic, and α-glucosidase inhibitory activities. In recent years, marine-derived, extremophilic, plant endophytic, and phytopathogenic fungi have emerged as important resources for diverse sorbicillinoids with unique skeletons. The recently revealed biological activities of sorbicillinoids discovered before 2016 are also described in this review.

## 1. Introduction

Sorbicillinoids are a family of fungal metabolites related to the hexaketide sorbicillin, and typically contain a sorbyl side chain in the structures with highly oxygenated frameworks [1,2]. According to their structural characteristics and biosynthesis, sorbicillinoids are divided into four groups: monomeric, dimeric, trimeric and hybrid sorbicillinoids [2]. Since sorbicillin (**1**) was first discovered from *Penicillium notatum* in 1948 [3], about 159 sorbicillinoids have been reported from fungi, especially those from genera *Penicillium* and *Trichoderma*.

Sorbicillinoids have potential pharmaceutical and agrochemical value as antimicrobial, antivirus, and anticancer agents, as well as pigments and food colorants. Sorbicillinoids and their biological activities have been well-reviewed before 2016 [1,2]. In 2011, Harned and Volp reviewed the structures of 62 sorbicillinoids [1]. Successively, 28 additional sorbicillinoids were reviewed by Meng et al. in 2016 [2]. Since then, dozens of new analogues have emerged.

In this mini-review, we focus on the recently identified structures of 69 sorbicillinoids along with their biological activities including newly revealed bioactivities of the sorbicillinoids discovered before 2016, in order to increase the diversity of identified sorbicillinoids as well as to speed up their applications.

## 2. Occurrence

**Figure 1:**
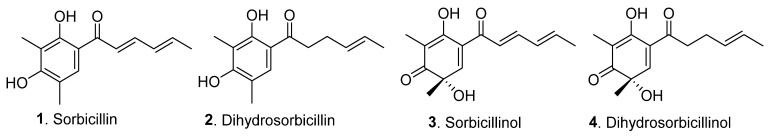
Basic structures of the monomeric sorbicillinoids (**1**–**4**).

**Figure 2:**
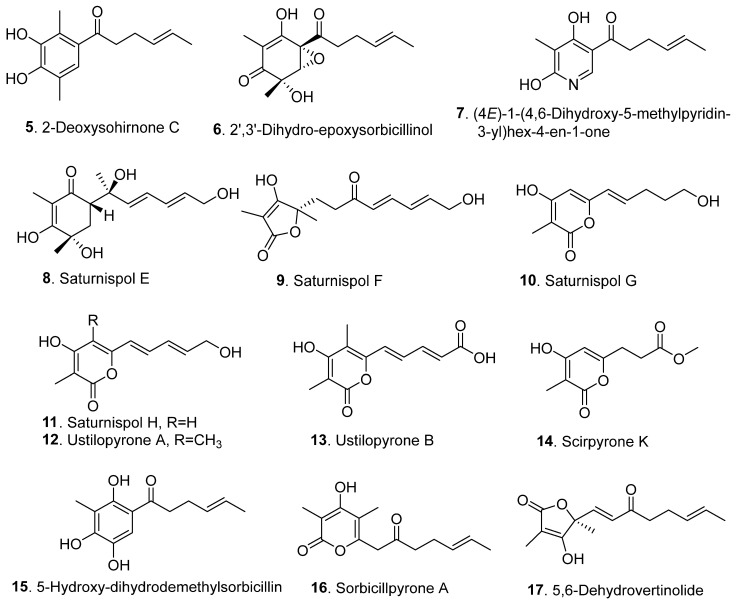

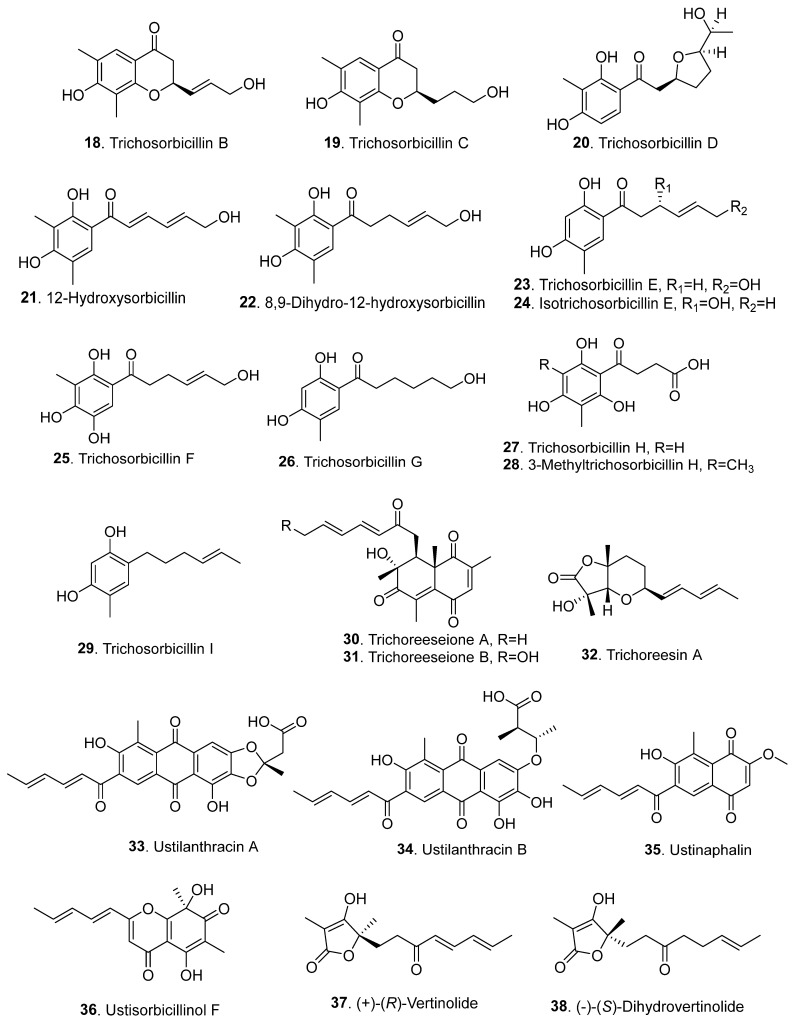
Structures of the monomeric sorbicillinoids (**5**–**38**) isolated from fungi.

**Figure 3:**
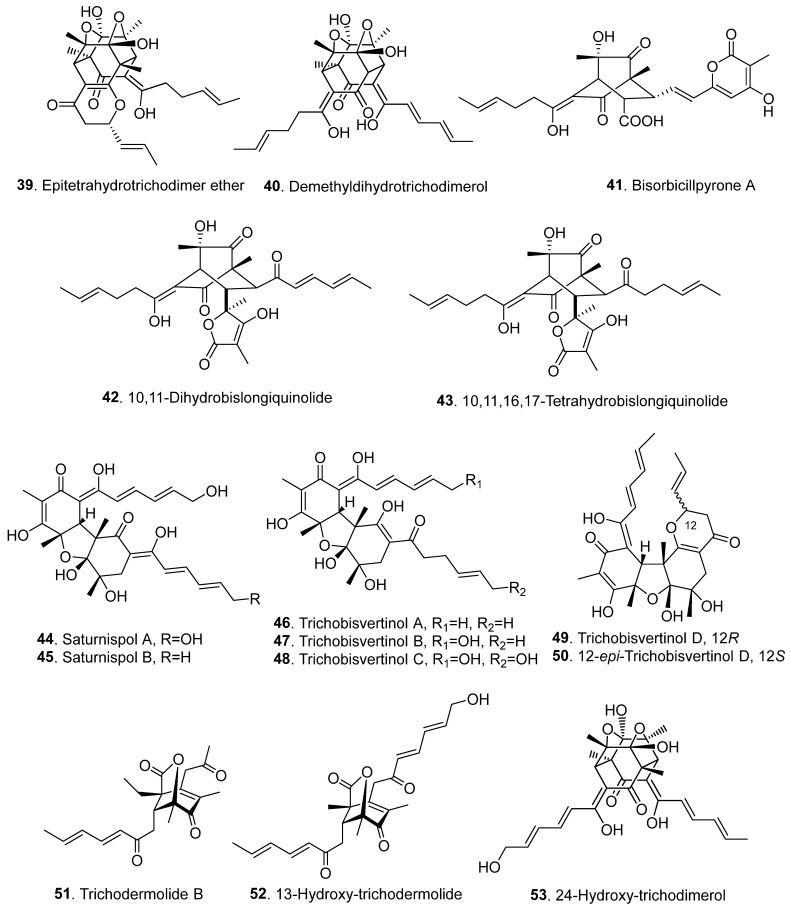

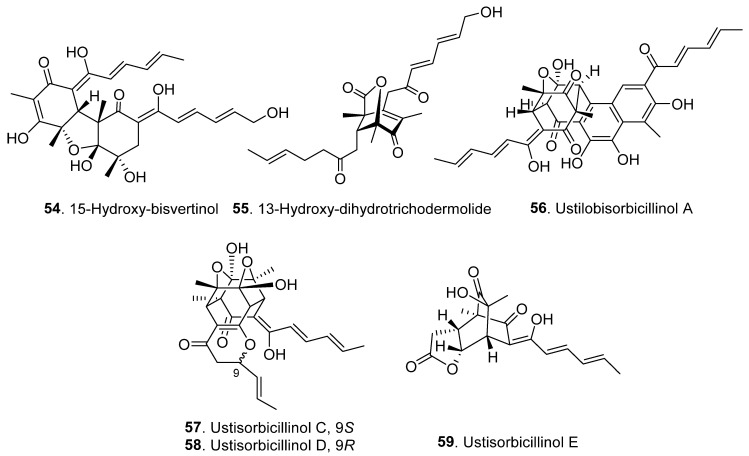
Structures of the bisorbicillinoids (**39**–**59**) isolated from fungi.

**Figure 4:**
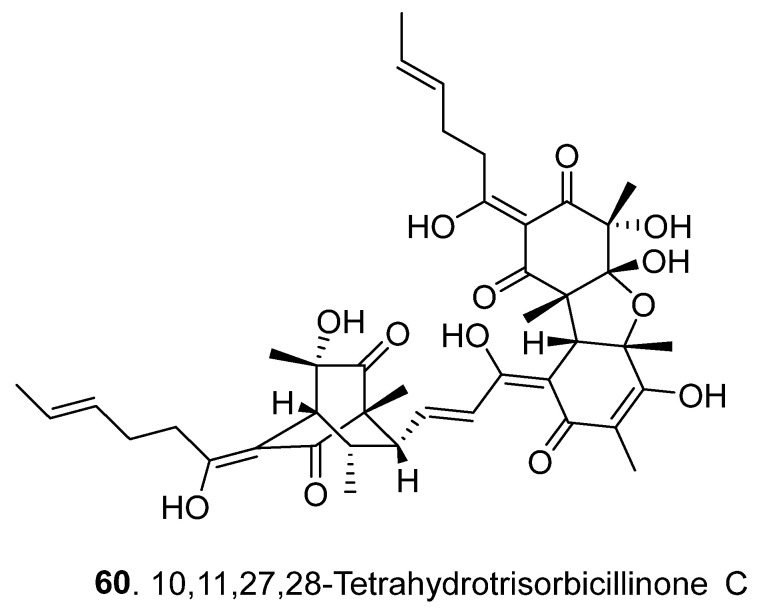
Structure of the trisorbicillinoid (**60**) isolated from fungi.

**Figure 5:**
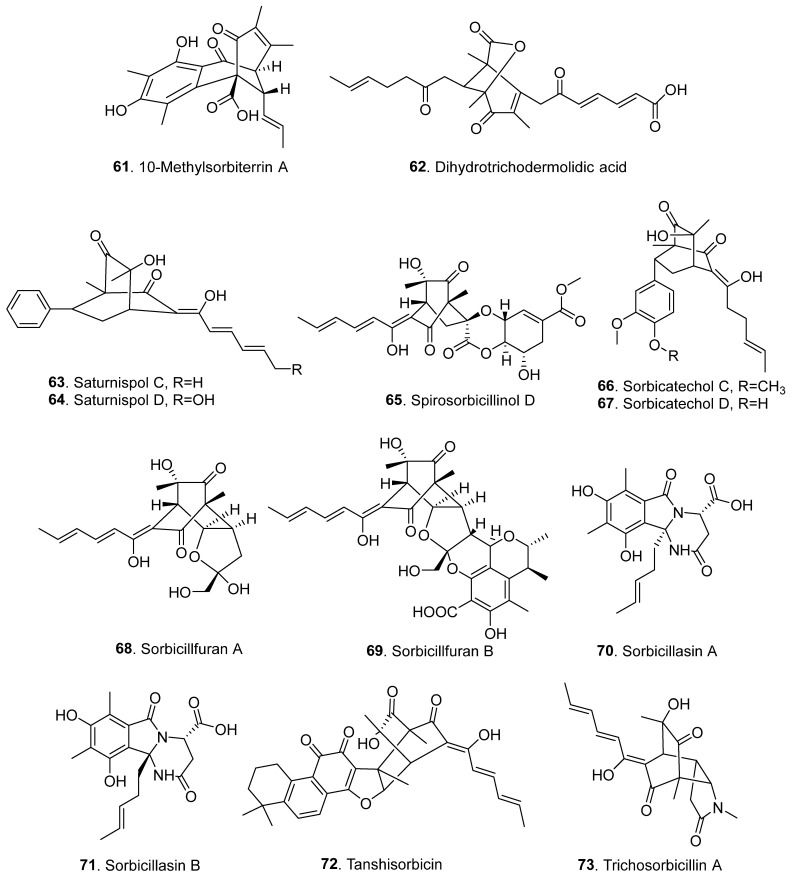
Structures of the hybrid sorbicillinoids (**61**–**73**) isolated from fungi.

Sorbicillinoids have a diverse distribution in fungi. In total, 69 sorbicillinoids have been isolated since 2016. They have mainly been found in plant endophytic, marine-derived, extremophilic, phytopathogenic fungi, and soil-derived fungi, mainly from the genera of *Acremonium*, *Aspergillus*, *Clonostachys*, *Penicillium*, *Ustilaginoidea,* and *Verticillium* [4,5]. All these fungi belong to the ascomycetes. The structures of sorbicillinoids are shown in Figure 1, Figure 2, Figure 3, Figure 4 and Figure 5.

### 2.1. Monomeric Sorbicillinoids

Sorbicillinoid monomers are the basic units of the sorbyl-containing metabolites catalyzed by polyketide synthases such as SorA and SorB [6]. The initial monomeric sorbicillinoid is sorbicillin (**1**), which is subsequently converted to dihydrosorbicillin (also called 2′,3′-dihydrosorbicillin, (**2**), sorbicillinol (**3**), dihydrosorbicillinol (also called 2′,3′-dihydrosorbicillinol, **4**), and other sorbicillinoids (Figure 1) [7]. The biosynthesis of the monomeric sorbicillinoids was revealed mainly based on genome research. An FAD-dependent monooxygenase encoding gene (*sorbC*) was cloned from *Penicillium chrysogenum* E01-10/3 and expressed as a soluble protein in *Escherichia coli*. The enzyme efficiently performed the oxidative dearomatization of sorbicillin (**1**) and dihydrosorbicillin (**2**) to produce sorbicillinol (**3**) and dihydrosorbicillinol (**4**), respectively [8].

Since 2016, thirty-four monomeric sorbicillinoids (Figure 2 and Table S1) have been isolated from fungi of the genera *Penicillium*, *Trichoderma*, *Ustilaginoidea*, *Phialocephala*, and *Clonostachys.* 2-deoxysohirnone C (**5**) was isolated from *Penicillium* sp. GD6 from the mangrove plant *Bruguiera gymnorrhiza* [9], and later isolated from *Penicillium* sp. SCSIO06871 from deep-sea sediment collected from the Indian Ocean [10].

2′,3′-dihydro-epoxysorbicillinol (**6**) was isolated as a new natural compound from *Trichoderma longibrachiatum* SFC100166, which was isolated from foreshore soil [11].

(4*E*)-1-(4,6-dihydroxy-5-methylpyridin-3-yl)hex-4-en-1-one (**7**) is a nitrogen-containing monomeric sorbicillinoid that was isolated from *Penicillium* sp. DM815 from the rhizosphere soil of a *Hibiscus tiliaceus* mangrove [12].

Four monomeric sorbicillinoids, namely saturnispols E (**8**), F (**9**), G (**10**) and H (**11**), were isolated from *Trichoderma saturnisporum* DI-IA from the marine sponge *Dictyonella incisa* collected at a depth of 10 m in Seferihisar Bay in Turkey [13]. Saturnispol H (**11**) is also named 5-demethylustilopyrone A (**11**), which was later isolated from the rice false smut pathogen *Ustilaginoidea virens* [14].

Both ustilopyrones A (**12**) and B (**13**), with pyrone structures, were isolated from rice false smut pathogen *Ustilaginoidea virens* [14]. Subsequently, ustilopyrone B (**13**) was re-isolated from *Penicillium* sp. SCSIO06871 from deep-sea sediment [10].

Scipyrone K (**14**), with a 3,4,6-trisubstituted α-pyrone structure, was isolated from the fungus *Phialocephala* sp. FL30r obtained from a deep seawater sample [15].

Three sorbicillinoids, namely 5-hydroxy-dihydrodemethylsorbicillin (**15**), sorbicillpyrone A (**16**), and 5,6-dehydrovertinolide (**17**), were isolated from *Penicillium* sp. SCSIO06871 from the deep-sea sediment [10].

Twelve monomeric sorbicillinoids including trichosorbicillins B (**18**), C (**19**), and D (**20**); 12-hydroxysorbicillin (**21**); 8,9-dihydro-12-hydroxysorbicillin (**22**); trichosorbicillin E (**23**); isotrichosorbicillin E (**24**); trichosorbicillins F (**25**), G (**26**), and H (**27**); 3-methyltrichosorbicillin H (**28**); and trichosorbicillin I (**29**) were isolated from marine-derived *Trichoderma reesei* 4670 associated with a sponge [16].

Trichoreeseiones A (**30**) and B (**31**) were isolated from an unidentified sponge-derived fungus *Trichoderma reesei* HN-2016-018. Both sorbicillinoids, with a characteristic naphthalene-trione ring, were first reported in the sorbicillinoid family [17].

Trichoreesin A (**32**) was the first bicyclic vertinolide derivative isolated from *Trichoderma reesei* Z56-8, an epiphytic fungus from the marine brown alga *Sargassum* sp. [18].

Ustilanthracins A (**33**) and B (**34**) were isolated from the rice false smut pathogen *Ustilaginoidea virens*. Both compounds share the same skeleton, but differ in the carboxyl-containing side chain, where dioxygenated butyric acid and 2-methyl-3-oxygenated butyric acid are found in ustilanthracins A (**33**) and B (**34**), respectively [19]. Both ustinaphthalin (**35**) and ustisorbicillinol F (**36**) were successively isolated from rice false smut pathogen *Ustilaginoidea virens* [14,19].

Vertinolides, with the presence of a γ-lactone terminus and a lack of any carbon rings, represent a class of degrading products of monomeric sorbicillinoids [20]. Three vertinolides, namely trichoreesin A (**32**), (+)-(*R*)-vertinolide (**37**), and (−)-(*S*)-dihydrovertinolide (**38**), have been isolated from fungi since 2016 [18,21,22]. (+)-(*R*)-vertinolide (**37**) is a new natural product isolated from *Trichoderma citrinoviride* from indoor air [21]. (*R*)-vertinolide (**37**) differs in stereochemistry from (*S*)-vertinolide isolated from *Verticillium intertextum* [23]. (−)-(*S*)-dihydrovertinolide (**38**) was isolated from the endophytic fungus *Clonostachys rosea* B5-2, which was isolated from the mangrove plant *Bruguiera gymnorrhiza*, collected in the coast of Santolo Garut Beach, West-Java, Indonesia [5,22].

### 2.2. Bisorbicillinoids

Bisorbicillinoids (also called dimeric sorbicillinoids) are formed by either an intermolecular Diels–Alder or Michael reaction of two monomeric sorbicillinoids [24]. Since 2016, twenty-one bisorbicillinoids have been isolated from fungi (Figure 3 and Table S2). These compounds are mainly distributed in the fungi genera *Penicillium, Trichoderma*, and *Ustilaginoidea*.

Three bisorbicillinoids, namely epitetrahydrotrichodimer ether (**39**), demethyldihydrotrichodimerol (**40**), and bisorbicillpyrone A (**41**), were isolated from *Penicillium* sp. SCSIO06871 from the deep-sea sediment. Among them, bisorbicillpyrone (**41**) is the first example of an α-pyrone-containing bisorbicillinoid [10].

Both 10,11-dihydrobislongiquinolide (**42**) and 10,11,16,17-tetrahydrobislongiquinolide (**43**) were produced by overexpression of the global regulator LaeA in the fungus *Penicillium dipodomyis* YJ-11 from a marine sediment sample collected in Jiaozhou Bay in Qingdao, China [25].

Saturnispols A (**44**) and B (**45**) were isolated from *Trichoderma saturnisporum* DI-IA from the marine sponge *Dictyonella incisa* collected in Seferihisar Bay in Turkey [13]. Saturnispols A (**44**) and B (**45**) are also named 15,24-dihydroxybisvertinol (**44**) and 24-hydroxybisvertinol (**45**), respectively. They were successively isolated from the marine-derived *Trichoderma reesei* 4670 from a sponge collected in Shantou, Guangdong, China [16]. Saturnispol B (**45**) was also isolated from an unidentified sponge-derived fungus *Trichoderma reesei* HN-2016-018 [17].

Five dimers, including trichobisvertinols A (**46**), B (**47**), C (**48**), and D (**49**), and 12-*epi*-trichobisvertinol D (**50**), were isolated from the marine-derived *Trichoderma reesei* 4670 from a sponge collected in Shantou, Guangdong, China [16]. Both trichobisvertinol D (**49**) and 12-*epi*-trichobisvertinol D (**50**) are epimeric to each other. Interestingly, they were isolated from *Ustilaginoidea virens* at the same time, and were named ustisorbicillinols A (**49**) and B (**50**), respectively [14].

Four dimeric sorbicillinoids, namely trichodermolide B (**51**), 13-hydroxy-trichodermolide (**52**), 24-hydroxy-trichodimerol (**53**), and 15-hydroxy-bisvertinol (**54**), were isolated from the sponge-derived fungus *Trichoderma reesei* HN-2016-018. Among them, trichodermolide B (**51**) and 13-hydroxy-trichodermolide (**52**) contain a unique bicycle [3.2.1] lactone skeleton. Trichodermolide B (**51**) with a propan-2-one moiety was firstly recorded in sorbicillinoid family [17]. 13-Hydroxy-dihydrotrichodermolide (**55**) is a structurally similar compound isolated from *Penicillium chrysogernum* 581F1 from the marine sponge *Theonella swinhoei* [26].

Ustilobisorbicillinol A (**56**) is a bisorbicillinoid featuring a unique cage structure that incorporates one sorbicillinol and one sorbyl-containing phenanthrenone unit. It was isolated from a culture of *Ustilaginoidea virens*, the rice false smut pathogen [19]. Three other bisorbicillinoids, namely ustisorbicillinols C (**57**), D (**58**), and E (**59**), were also isolated from *Ustilaginoidea virens*. Both ustisorbicillinols C (**57**) and D (**58**) are epimeric to each other [14].

### 2.3. Trisorbicillinoids

Trisorbicillinoids (or called trimeric sorbicillinoids) are formed by either an intermolecular Diels–Alder or Michael reaction of three monomeric sorbicillinoids [24]. Only one trisorbicillinoid, 10,11,27,28-tetrahydrotrisorbicillinone C (**60**), has been isolated from *Penicillium chrysogernum* 581F1 from the marine sponge *Theonella swinhoei* since 2016 (Figure 4) [26].

### 2.4. Hybrid Sorbicillinoids

Hybrid sorbicillinoids are derived from either an asymmetrical Diels–Alder reaction of a monomeric sorbicillinoid diene and a second non-sorbicillinoid dienophile [24]. About 13 hybrid sorbicillinoids have been isolated from fungi since 2016 (Figure 5 and Table S3). Two hybrids, 10-methylsorbiterrin (**61**) and dihydrotrichodermolidic acid (**62**), were isolated from *Penicillium* sp. SCSIO06871 from the deep-sea sediment [10].

Both saturnispols C (**63**) and D (**64**) were isolated from *Trichoderma saturnisporum* DI-IA from the marine sponge *Dictyonella incisa* collected in Seferihisar Bay in Turkey. Biogenetically, it was proposed that the [4+2] Diels–Alder cycloaddition of sorbicillinol with a phenylethylene generated saturnispol C (**63**), followed by hydroxylation, to yield saturnispol D (**64**) [13].

Spirosorbicillinol D (**65**) is a hybrid sorbicillinoid from *Trichoderma longibrachiatum* SFC100166 isolated from foreshore soil [11].

Sorbicatechols C (**66**) and D (**67**) were isolated from *Penicillium allii-sativi* from deep-sea water [27].

Sorbicillfurans A (**68**) and B (**69**) were isolated from the static culture of the fungus *Penicillium citrinum* SCSIO41402, which was isolated from a marine alga *Coelarthrum* sp. collected in Yongxing Island, South China Sea. Both compounds possess a tetrahydrofuran unit. It was suggested that both sorbicillfurans A (**68**) and B (**69**) are derived from the precursor sorbicillinol added with furfuryl alcohol by a Diels–Alder (DA) reaction, followed by the oxidization modification to yield sorbicillfuran A (**68**), and by another DA cycloaddition reaction to generate sorbicillfuran B (**69**) [28].

Two nitrogen-containing sorbicillinoids with hexahydropyrimido [2,1-*a*] isoindole moiety named sorbicillasins A (**70**) and B (**71**) were isolated from the deep-sea fungus *Phialocephala* sp. FL30r obtained from an underwater sample. Sorbicillasins A (**70**) and B (**71**) are probably formed by adding a whole molecule of L-asparagine to 2′,3′-dihydrosorbicillin via sequential intermolecular/intramolecular nucleophilic reactions [15].

When tanshinone IIA was fed to the fermentation cultures of sorbcillinol-producing fungus *Hypocrea* sp., the hybrid sorbicillinoid produced was tanshisorbicin (**72**), which is considered a [4+2] cycloaddition adduct between tanshinone IIA and sorbicillinol (**3**) [29].

Trichosorbicillin A (**73**) is a nitrogen-containing sorbicillinoid isolated from the marine-derived *Trichoderma reesei* 4670 from a sponge collected in Shantou, Guangdong, China. It was hypothesized to arise from a net [4+2] cycloaddition or double Michael reaction between sorbicillinol (**3**) and 1-methyl-1,3-dihydro-2*H*-pyrrol-2-one [16].

## 3. Biological Activities

**Figure 6:**
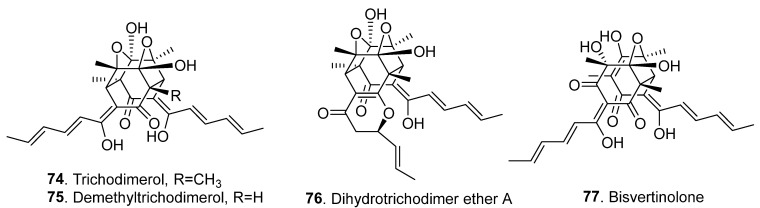

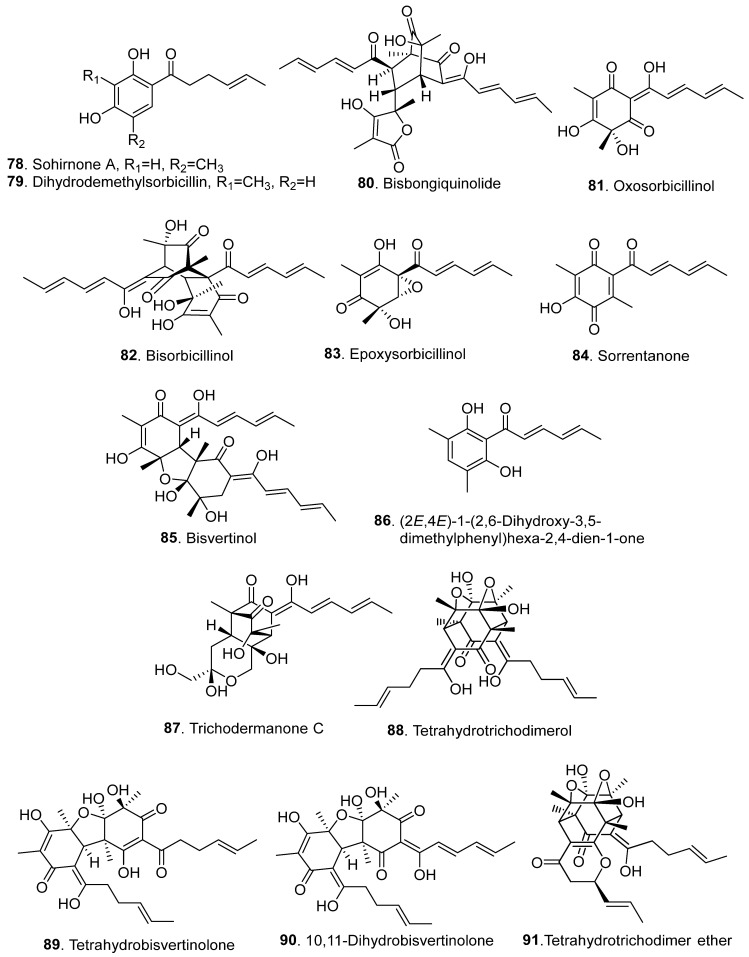
Structures of some sorbicillinoids (**74**–**91**) discovered before 2016 with newly revealed biological activities.

The recently isolated sorbicillinoids mainly display cytotoxic, antibacterial, antifungal, anti-inflammatory, phytotoxic, and α-glucosidase inhibitory activities (Tables S4–S10). The structures of some sorbicillinoids (**74**–**91**) discovered before 2016 with newly revealed biological activities are shown in Figure 6.

### 3.1. Cytotoxic Activity

Some recently revealed sorbicillinoids displayed obviously cytotoxic activities (Table S4). Sorbicatechol D (**67**) and sorbicillin (**1**) were screened to show antiproliferative activity on HT-29 tumor cells in a dose-dependent manner. The mechanism investigation uncovered that they can significantly induce cell cycle G2–M phase arrest by increasing the protein levels of p-H3 and cyclin B1 [27]. Sorbicillin (**1**) was once again isolated from the culture broth of the fungus *Penicillium decumbens* from a limestone soil. It exhibited selective cytotoxic activity against the human hepatocellular carcinoma (QGY-7703) cells with an IC_50_ value of 32.5 μM [30]. Similar cytotoxic activity results of sorbicillin (**1**) have been reported previously [31,32,33].

Sorbicillfuran B (**69**) showed weak cytotoxic activity against human leukemia cell line HL-60 cells with an IC_50_ value of 9.6 μM [28]. Five cytotoxic bisorbicillinoids, namely ustilobisorbicillinol A (**56**), trichodimerol (**74**), demethyltrichodimerol (**75**), dihydrotrichodimer ether (**76**), and bisvertinolone (**77**), were isolated from the rice false smut pathogen *Ustilaginlidea virens* [14,19]. Among them, trichodimerol (**74**), demethyltrichodimerol (**75**), dihydrotrichodimer ether A (**76**), and bisvertinolone (**77**) showed moderate cytotoxic activities on human carcinoma cells with IC_50_ values of 8.83–74.7 μM [14]. Ustilobisorbicillinol A (**56**) showed notable cytotoxicity against the five tested tumor cell lines, with IC_50_ values in the range of 4.48–18.6 μM. It was further tested for its influence on cell-cycle progression with the gastric cancer cell line BGC823. Interestingly, it markedly induced G0/G1- and G2/M-phase cell-cycle arrest. Ustilobisorbicillinol A (**56**) was also investigated for its effect on apoptosis in BGC823 cells, as cell shrinkage and detached from culture surface was observed after treatment with ustilobisorbicillinol A (**56**). The apoptotic rate of BGC823 cells was examined using flow cytometry. Compared to the control group, treatment with ustilobisorbicillinol A (**56**) at 9 μM for 48 h induced significant apoptosis incidence in BGC823 cells (74.7%). Moreover, treatment with ustilobisorbicillinol A (**56**) altered the expression levels of cleaved caspase-3 and PARP, suggesting the caspase-mediated apoptotic pathway is involved in the induced apoptosis of BGC823 cells [19].

24-hydroxy-trichodimerol (**53**) displayed cytotoxic activities against human tumor cells (A549, MCF-7, and HCT116) with IC_50_ values of 5.1, 9.5, and 13.7 mM, respectively [17].

### 3.2. Antibacterial Activity

Due to the long-term use of some antibiotics, the bacterial or fungal pathogens easily develop drug resistance, and it is necessary to look for new alternatives. Some sorbicillinoids exhibited obvious antibacterial activities, showing their potential as the antimicrobials (Table S5). Two monomeric sorbicillinoids, saturnispols F (**9**) and H (**11**), showed significant antibacterial activity. Saturnispol F (**9**) displayed inhibition of bacteria with minimum inhibitory concentration (MIC) values of 3.32 μg/mL against *Staphylococcus aureus*, 1.63 μg/mL against vancomycin-resistant *Enterococci faecalis* (VRE), 6.65 μg/mL against *Pseudomonas aeruginosa*, and 6.65 μg/mL against *Klebsiella pneumoniae*. Saturnispol H (**11**) displayed inhibition of bacteria with MIC values of 12.9 μg/mL against vancomycin-resistant *Enterococci faecalis* and 12.9 μg/mL against *Bacillus subtilis* [13].

Both sohirnone A (**78**) and dihydrodemethylsorbicillin (**79**) exhibited significant antibacterial activities against *Staphylococcus aureus* with MIC values of 10.0 μg/mL and 5.0 μg/mL, respectively [10].

Five sorbicillinoids ustisorbicillinol B (or 12-*epi*-trichobisvertinol D (**50)**), demethyltrichodimerol (**75**), dihydrotrichodimer ether A (**76**), bisvertinolone (**77**), and oxosorbicillinol (**81**) from *Ustilaginoidea virens* showed antibacterial activities against six human/plant pathogenic bacteria. Among them, bisvertinolone (**77**) was the most effective [14]. A similar antibacterial activity of oxosorbicillinol (**81**) was reported previously [34]. Bisvertinolone (**77**), isolated from *Aspergillus protuberus* MUT3638, was also previously reported to exhibit significant activity against *Staphylococcus aureus* with an MIC value of 30 μg/mL [35]. Two bisorbicillinoids, bisvertinolone (**77**) and bislongiquinolide *saturnisporum* (**80**), were screened to show antibacterial activities against *Pseudomonas lachrymans* with MIC values of 3.13 and 1.56 μM, respectively, and against *Escherichia coli* with MIC values of 6.25 and 12.5 μM, respectively [36].

Tanshisorbicin (**72**) showed obvious antibacterial activity on *Mycobacterium bovis*, *Staphylococcus aureus* (ATCC 6538), methicillin-resistant *Staphylococcus aureus* (MRSA), and *Bacillus subtilis* (ATCC 6633). The anti-MRSA activity of tanshisorbicin (**72**) was found to be significantly higher than that of tanshinone IIA [29].

Antibacterial mechanisms showed that sorbicillinoids could generate singlet oxygen (^1^O_2_) under UV light irradiation and ultimately displayed photoinactivation activity on Gram-positive bacteria including *Staphylococcus aureus*, *Bacillus subtilis*, and *Micrococcus luteus*, but not Gram-negative ones such as *Escherichia coli* and *Proteus vulgaris*, showing their potential as photosensitizers for antimicrobial photodynamic therapy using a nontoxic dose of UV irradiation [37].

### 3.3. Antifungal Activity

Some recently discovered sorbicillinoids were screened for antifungal activities (Table S6). Sorbicillin (**1**) displayed antifungal activity toward *Candida albicans* Y0109 with an MIC value of 50 μM [30].

Bisvertinolone (**77**), oxosorbicillinol (**81**), bisorbicillinol (**82**), and epoxysorbicillinol (**83**) from *Trichoderma longibrachiatum* SFC100166 were screened for antifungal activity on phytopathogenic fungi *Cladosporium coccodes, Colletotrichum coccodes*, *Cylindrocarpon destructans*, *Magnaporthe oyrzae,* and *Phytopathora infestans*, with MIC values ranging from 6.3 to 100 μg/mL. When tomato plants were treated with the above compounds (**77**,**81**–**83**), bisvertinolone (**77**) strongly reduced the development of tomato late blight disease compared to the untreated control [11].

Demethyltrichodimerol (**75**), bisvertinolone (**77**), and oxosorbicillinol (**81**) displayed moderate antifungal activities by inhibiting the spore germination of rice blast pathogen *Magnaporthe oryzae*. Among them, bisvertinolone (**77**) was the most effective sorbicillinoid [14].

### 3.4. Anti-Inflammatory Activity

Inflammation is a common response of the human body to injuries caused by microbial pathogens, trauma, or toxic compounds. Bioactive metabolites produced by fungi have received considerable attention as new therapeutic agents [38]. Many sorbicillinoids were screened for anti-inflammatory activities and their potential use in the treatment of inflammatory diseases (Table S7). Trichodimerol (**74**) and sorrentanone (**84**) were isolated from the endophytic fungus *Trichoderma* sp. Xy24 from the mangrove plant *Xylocarpus granatum*. Both compounds displayed anti-inflammatory activity by inhibiting LPS-induced NO production in BV2 microglia cells, with the inhibitory rates of 75.1% and 100.0% at 10 μM, respectively, much more potent than the positive control curcumin [39].

Eighteen mono- and dimeric sorbicillinoids, including trichosorbicillin B (**18**), trichosorbicillin C (**19**), 12-hydroxysorbicillin (**21**), 8,9-dihydro-12-hydroxysorbicillin (**22**), trichosorbicillin E (**23**), isotrichosorbicillin E (**24**), trichosorbicillin F (**25**), trichosorbicilin I (**29**), 24-hydroxybisvertinol (also named saturnispol B, **45**), trichobisvertinol A (**46**), trichobisvertinol B (**47**), trichobisvertinol C (**48**), trichobisvertinol D (**49**), 12-*epi*-trichobisvertinol D (**50**), sohirnone A (**78**), bisvertinol (**85**), 2′,3′-dihydrosorbicillin (also called dihydrosorbicillin, **2**), and (2*E*,4*E*)-1-(2,6-Dihydroxy-3,5-dimethylphenyl)hexa-2,4-dien-1-one (**86**) from the sponge-derived fungus *Trichoderma reesei* 4670, were systematically screened for potent anti-inflammatory activity by inhibiting the production of NO in RAW264.7 cells activated by lipopolysaccharide, with IC_50_ values in the range of 0.94 to 38 μM. The structure−activity relationship analysis indicated that the anti-inflammatory activities of the sorbicillinoids mainly depend on the structural types and the functional groups of the sorbyl side chain [16].

Trichodermanone C (**87**) is a hybrid sorbicillinoid showing an anti-inflammatory activity with inhibition of nitrite levels in lipopolysaccharide (LPS)-stimulated J774A.1 macrophages [40].

Epitetrahydrotrichodimer ether (**39**) and tetrahydrotrichodimerol (**88**) are two dimeric sorbicillinoids isolated from *Penicillium* sp. DM815 from the rhizosphere soil of mangrove *Hibiscus tiliaceus* that significantly reduced the level of NO produced by inducible nitric oxide synthase (iNOS) [12].

### 3.5. Phytotoxic Activity

Plant pathogenic and endophytic fungi usually produce metabolites poisonous to their host plants. These phytotoxic metabolites from fungi are called phytotoxins [41]. It is considered that the amounts of phytotoxins produced by the endophytic fungi are much lower than those of the phytopathogenic fungi [42].

Four sorbicillinoids (Table S8), namely trichodimerol (**74**), demethyltrichodimerol (**75**), bisvertinolone (**77**), and bislongiquinolide (also named trichotetronine, **80**) from rice false smut pathogen *Ustilaginoidea virens,* showed phytotoxic activity by inhibiting radicle and germ elongation of rice and lettuce seedlings, with bisvertinolone (**77**) displaying the strongest inhibition. These phytotoxic sorbicillinoids might play an important role in the development of rice false smut symptoms [14].

(−)-(*S*)-dihydrovertinolide (**38**) inhibited the shoot growth by 23% and root growth by 65% of lettuce (*Lactuca sativa*) seedlings [22].

### 3.6. α-Glucosidase Inhibitory Activity

Diabetes is considered as one of the biggest current health crises. Controlling carbohydrate digestibility by inhibiting starch digestive enzyme (i.e., α-amylase and α-glucosidase) activities is an efficient strategy to control postprandial hyperglycemia [43]. Some sorbicillinoids have been screened for their α–glucosidase inhibitory activity (Table S9).

Six sorbicillinoids, including 5-hydroxy-dihydrodemethylsorbicillin (**15**), bisorbicillpyrone A (**41**), dihydrodemethylsorbicillin (**79**), tetrahydrotrichodimerol (**88**), tetrahydrobisvertinolone (**89**), and 10,11-dihydrobisvertinolone (**90**), exhibited α-glucosidase inhibitory activity, with IC_50_ values ranging from 115.8 to 208.5 μM. Among these, 5-hydroxy-dihydrodemethylsorbicillin (**15**) showed the strongest inhibitory activity against α-glucosidase with an IC_50_ value of 36.0 μM, stronger than that of acarbose [10].

2′,3′-dihydrosorbicillin (**2**), which was isolated from the fungus *Aspergillus* sp. HNWSW-20 from Chinese agarwood (*Aquilaria sinensis*), showed α-glucosidase inhibitory activity [44].

### 3.7. Other Biological Activities

Other biological activities of the sorbicillinoids recently revealed from fungi mainly include antiallergic, antioxidant, neuroprotective and neuritogenic, antihuman-immunodeficiency-virus (HIV), and antimicroalgal activities, as well as inhibitory activities against acetylcholinesterase (AChE) and protein tyrosine phosphatase 1B (Table S10).

Bisorbicillinol (**82**) is a bisorbicillinoid previously isolated from a few fungi such as *Trichoderma* sp. USF-2690 [45], *Trichoderma* sp. f-13 [31], and *Penicillium notatum* [34]. Bisorbicillinol (**82**) from *Trichoderma* sp. USF2690 was found to be an inhibitor of β-hexosaminidase release and tumor necrosis factor (TNF)-α, and 9nterleukin (IL)-4 secretion from rat basophilic leukemia (RBL-2H3) cells, with IC_50_ values of 2.8, 2.9, and 2.8 μM, respectively. The results showed that the inhibitory mechanism of β-hexosaminidase release and TNF-α secretion involve inhibition of Lyn, a tyrosine kinase. This indicated that bisorbicillinol (**82**) should be a candidate antiallergic agent [46].

Scipyrone K (**14**), isolated from the fungus *Phialocephala* sp. FL30r obtained from a deep seawater sample, exhibited weak radical scavenging activity against 2,2-diphenyl-1-picrylhydrazyl (DPPH) with an IC_50_ value of 27.9 μM [15].

Sorbicillin (**1**) was proven to have neuroprotective and neuritogenic activity on PC-12 Adh cells of the 6-hydroxydopamine-induced Parkinson’s disease cell model at 1 and 10 μg/mL. The water fraction of halotolerant *Penicillium flavigenum* isolated from Salt Lake in Konya, Turkey, also showed similar activity. The water extract was revealed to contain sorbicillin-like active metabolites by LC-MS compared to a sorbicillin (**1**) standard [47]. Sorbicillin (**1**) and 2′,3′-dihydrosorbicillin (**2**) showed acetylcholinesterase inhibitory activities with inhibition rates of 15.47% and 1.78%, respectively, at a concentration of 50 μg/mL [44].

At a concentration of 40 μM, both 2′,3′-dihydrosorbicillin (**2**) and sohirnone A (**78**) exhibited moderate inhibitory activity of protein tyrosine phosphatase 1B (PTP1B) with inhibitory ratios of 10.58% and 8.47%, respectively, to show their antidiabetic potential [48].

Sorrentanone (**84**) showed a significant inhibitory effect of HIV-1 virus with an IC_50_ value of 4.7 μM, so is worthy of further investigation as a lead anti-HIV compound [38].

Glucagon-like peptide-1 (GLP-1), a gut incretin hormone that stimulates insulin and inhibits glucagon secretion on pancreatic β-cells and α-cells, is considered a target protein related to diabetes. Eukaryotic elongation factor-2 kinase (eEF2K) is a potential therapeutic target for cancer. Both 13-hydroxy-dihydrotrichodermolide (**55**) and 10,11,27,28-tetrahydrotrisorbicillinone C (**60**) displayed high affinities to target proteins GLP-1R and eEF2K with K_d_ values of 0.0285 and 0.0162 μM for GLP-1R, and 0.118 and 0.0746 μM for eEF2K, respectively. These findings indicate that 13-hydroxy-dihydrotrichodermolide (**55**) and 10,11,27,28-tetrahydrotrisorbicillinone C (**60**) are promising new drug candidates for diabetes and cancer treatment [26].

Both tetrahydrobisvertinolone (**89**) and tetrahydrotrichodimer ether (**91**) exhibited weak acetylcholinesterase (AChE) inhibitory activity with 51.1% and 55.1% inhibitions at a concentration of 50 μg/mL, respectively [10].

Trichoreesin A (**32**) showed antimicroalgal activity against the marine algae *Chattonella marina*, *Heterosigma akashiwo*, and *Prorocentrum donghaiense* with IC_50_ values of 13, 29, and 2.8 μg/mL, respectively [18].

## 4. Conclusions

From 2016 to 2021, 69 new sorbicillinoids were isolated from fungi. Mainly belonging to the monomeric and dimeric sorbicillinoids, some sorbicillinoids have special structures such as ustilobisorbicillinol A (**56**) [19], and sorbicillasins A (**70**) and B (**71**) [15], increasing their diversity. The majority of sorbicillinoids were reported from the fungi genera of *Acremonium*, *Penicillium*, *Trichoderma*, and *Ustilaginoidea*. This provides a basis for fungal chemotaxonomy, which should be further studied in detail. It is worth mentioning that 21 sorbicillinoids were firstly isolated from the rice false smut pathogen *Ustilaginoidea virens* [14,19], which can produce many types of bioactive secondary metabolites [49,50,51,52,53,54,55,56,57,58]. Some sorbicillinoids exhibited cytotoxic (Table S4), antibacterial (Table S5), antifungal (Table S6), anti-inflammatory (Table S7), phytotoxic (Table S8), and α-glucosidase-inhibitory (Table S9) and PTP1B-inhibitory activities (Table S10). They may be utilized as pigments and food colorants as well. Due to the limitation of activity screening models by each research group, many sorbicillinoids need to be further screened for their biological activities. Furthermore, the comparative investigations on the biological activities of sorbicillinoids and other classes of compounds along with their action mechanisms need to be further conducted [59,60,61]. In recent years, more and more new members of sorbicillinoids have been revealed from plant endophytic, marine-derived, extremophilic, phytopathogenic, and soil-derived fungi. All these sorbicillinoids may be rich resources of biologically active substances with significant pharmaceutical, food colorant, and agricultural value [2].

Fungal sorbicillinoids were studied extensively from 2016 to 2021. Apart from the discovery of new sorbicillinoids and clarification of their biological activities and action mechanisms, other related studies include biosynthetic gene clusters [6], biosynthetic pathways and their related enzymes [5,24,62,63,64,65], relevant regulatory mechanisms [7,25,66,67,68], biochemical engineering to increase the production of sorbicillinoids [59], chemoenzymatic synthesis [69], development of chemical synthesis methods [70], and applications of sorbicillinoids in the agriculture, pharmaceutical, and food industries [37,60,61]. Among them, the most promising is clarification of the Diels–Alder reactions during the biosynthesis of sorbicillinoids. Through co-expression of *sorA*, *sorB*, *sorC*, and *sorD* from *Trichoderma reesei* QM6a, the biosynthetic pathway to epoxysorbicillinol and dimeric sorbicillinoids resembling Diels–Alder-like and Michael-addition-like products was reconstituted in *Aspergillus oryzae* NSAR1 [24].

## Data Availability

Not applicable.

## Supplementary Materials

The following supporting information can be downloaded at: https://www.mdpi.com/article/10.3390/jof8010062/s1, Table S1: Occurrence of the monomeric sorbicillinoids (**5**–**38**) in fungi; Table S2: Occurrence of the bisorbicillinoids (**39**–**59**) in fungi; Table S3: Occurrence of the hybrid sorbicillinoids (**61**–**73**) in fungi; Table S4: Cytotoxic activity of the screened sorbicillinoids in fungi; Table S5: Antibacterial activity of the sorbicillinoids screened from fungi; Table S6: Antifungal activity of the sorbicillinoids screened from fungi; Table S7: Anti-inflammatory activity of the sorbicillinoids screened from fungi; Table S8: Phytotoxic activity of the sorbicillinoids screened from fungi; Table S9: α-Glucosidase inhibitory activity of the sorbicillinoids screened from fungi; Table S10: Other biological activities of the sorbicillinoids screened from fungi.
