# Supplementary material for: Recent Advances in Sorbicillinoids from Fungi and Their Bioactivities (Covering 2016–2021)
Source: J Fungi (Basel). 2022 Jan 7;8(1):62. doi: 10.3390/jof8010062 (PMC8779745; doi:10.3390/jof8010062)
Supplement: Supplementary file 1 [file jof-08-00062-s001.zip › jof-1541002-supplementary.pdf]

Supplementary Materials: Recent Advances in Sorbicillinoids from Fungi and Their Bioactivities (Covering 2016-2021)

Xuwen Hou, Xuping Zhang, Mengyao Xue, Zhitong Zhao, Huizhen Zhang, Dan Xu, Daowan Lai and Ligang Zhou *

State Key Laboratory of Agrobiotechnology, Department of Plant Pathology, College of Plant Protection, China Agricultural University, Beijing 100193, China; xwhou@cau.edu.cn (X.H.); zhangxuping5@cau.edu.cn (X.Z.); mengyaoxue@cau.edu.cn (M.X.); zhitongzhao@cau.edu.cn (Z.Z.); huizhenzhang@cau.edu.cn (H.Z.); cauxudan@cau.edu.cn (D.X.); dwlai@cau.edu.cn (D.L.)

* Correspondence: lgzhou@cau.edu.cn; Tel.: +86-10-6273-1199

Contents

Table S1. Occurrence of the monomeric sorbicillinoids (**5**–**38**) in fungi. 2

Table S2. Occurrence of the bisorbicillinoids (**39**–**59**) in fungi. 3

Table S3. Occurrence of the hybrid sorbicillinoids (**61**-**73**) in fungi. 4

Table S4. Cytotoxic activity of the screened sorbicillinoids from fungi 4

Table S5. Antibacterial activity of the screened sorbicillinoids from fungi. 5

Table S6. Antifungal activity of the screened sorbicillinoids from fungi. 5

Table S7. Anti-inflammatory activity of the screened sorbicillinoids from fungi. 6

Table S8. Phytotoxic activity of the screened sorbicillinoids from fungi 6

Table S9. α–Glucosidase inhibitory activity of the screened sorbicillinoids from fungi. 7

Table S10. Other biological activities of the screened sorbicillinoids from fungi. 7

Supplementary References 7

Table S1. Occurrence of the monomeric sorbicillinoids (**5**–**38**) in fungi.

Sorbicillinoid	Fungus and its Origin	Ref.
2-Deoxysohirnone C (**5**)	*Penicillium* sp. GD6 from mangrove *Bruguiera gymnorrhiza*	[1]
	Penicillium sp. SCSIO06871 from the deep-sea sediment	[2]
2′,3′-Dihydro-epoxysorbicillinol (**6**)	*Trichoderma longibrachiatum* SFC100166 isolated from the foreshore soil	[3]
(4*E*)-1-(4,6-Dihydroxy-5-methylpyridin-3-yl)hex-4-en-1-one (**7**)	*Penicillium* sp. DM815 from the rhizosphere soil of a mangrove *Hibiscus tiliaceus*	[4]
Saturnispol E (**8**)	*Trichoderma saturnisporum* DI-IA from the marine sponge *Dictyonella incisa*	[5]
Saturnispol F (**9**)	*Trichoderma saturnisporum* DI-IA from the marine sponge *Dictyonella incisa*	[5]
Saturnispol G (**10**)	*Trichoderma saturnisporum* DI-IA from the marine sponge *Dictyonella incisa*	[5]
Saturnispol H = 5-Demethylustilopyrone A (**11**)	*Trichoderma saturnisporum* DI-IA from the marine sponge *Dictyonella incisa*	[5]
	Rice false smut pathogen *Ustilaginoidea virens*	[6]
Ustilopyrone A (**12**)	Rice false smut pathogen *Ustilaginoidea virens*	[6]
Ustilopyrone B (**13**)	Rice false smut pathogen *Ustilaginoidea virens*	[6]
	Penicillium sp. SCSIO06871 from the deep-sea sediment	[2]
Scipyrone K (**14**)	Deep-sea-derived fungus *Phialocephala* sp. FL30r	[7]
5-Hydroxy-dihydrodemethylsorbicillin (**15**)	*Penicillium* sp. SCSIO06871 from the deep-sea sediment	[2]
Sorbicillpyrone A (**16**)	*Penicillium* sp. SCSIO06871 from the deep-sea sediment	[2]
5,6-Dehydrovertinolide (**17**)	*Penicillium* sp. SCSIO06871 from the deep-sea sediment	[2]
Trichosorbicillin B (**18**)	Marine-derived *Trichoderma reesei* 4670 from a sponge	[8]
Trichosorbicillin C (**19**)	Marine-derived *Trichoderma reesei* 4670 from a sponge	[8]
Trichosorbicillin D (**20**)	Marine-derived *Trichoderma reesei* 4670 from a sponge	[8]
12-Hydroxysorbicillin (**21**)	Marine-derived *Trichoderma reesei* 4670 from a sponge	[8]
8,9-Dihydro-12-hydroxysorbicillin (**22**)	Marine-derived *Trichoderma reesei* 4670 from a sponge	[8]
Trichosorbicillin E (**23**)	Marine-derived *Trichoderma reesei* 4670 from a sponge	[8]
Isotrichosorbicillin E (**24**)	Marine-derived *Trichoderma reesei* 4670 from a sponge	[8]
Trichosorbicillin F (**25**)	Marine-derived *Trichoderma reesei* 4670 from a sponge	[8]
Trichosorbicillin G (**26**)	Marine-derived *Trichoderma reesei* 4670 from a sponge	[8]
Trichosorbicillin H (**27**)	Marine-derived *Trichoderma reesei* 4670 from a sponge	[8]
3-Methyltrichosorbicillin H (**28**)	Marine-derived *Tricoderma reesei* 4670 from a sponge	[8]
Trichosorbicillin I (**29**)	Marine-derived *Trichoderma reesei* 4670 from a sponge	[8]
Trichoreeseione A (**30**)	Marine derived *Trichoderma reesei* from a sponge	[9]
Trichoreeseione B (**31**)	Marine derived *Trichoderma reesei* from a sponge	[9]
Trichoreesin A (**32**)	Epiphytic fungus *Trichoderma reesi* Z56-8 from the marine brown alga S*argassum* sp.	[10]
Ustilanthracin A (**33**)	Rice false smut pathogen *Ustilaginoidea virens*	[11]
Ustilanthracin B (**34**)	Rice false smut pathogen *Ustilaginoidea virens*	[11]

Ustinaphthalin (**35**)	Rice false smut pathogen *Ustilaginoidea virens*	[11]
Ustisorbicillinol F (**36**)	Rice false smut pathogen *Ustilaginoidea virens*	[6]
(+)-(*R*)-Vertinolide (**37**)	*Trichoderma citrinoviride* from indoor air	[12]
(-)-(*S*)-Dihydrovertinolide (**38**)	Endophytic fungus *Clonostachys rosea* B5-2 from *Bruguiera gymnorrhiza*	[13]

Table S2. Occurrence of the bisorbicillinoids (**39**–**59**) in fungi.

Sorbicillinoid	Fungus and its Origin	Ref.
Epitetrahydrotrichodimer ether (**39**)	*Penicillium* sp. SCSIO06871 from the deep-sea sediment	[2]
Demethyldihydrotrichodimerol (**40**)	*Penicillium* sp. SCSIO06871 from the deep-sea sediment	[2]
Bisorbicillpyrone A (**41**)	*Penicillium* sp. SCSIO06871 from the deep-sea sediment	[2]
10,11-Dihydrobislongiquinolide (**42**)	*Penicillium dipodomyis* YJ-11 from a marine sediment	[14]
10,11,16,17-Tetrahydrobislongiquinolide (**43**)	*Penicillium dipodomyis* YJ-11 from a marine sediment	[14]
Saturnispol A = 15,24-Dihydroxybisvertinol (**44**)	*Trichoderma saturnisporum* DI-IA from the marine sponge *Dictyonella incisa*	[5]
	Marine-derived *Trichoderma reesei* 4670 from a sponge	[8]
Saturnispol B = 24-Hydroxybisvertinol (**45**)	*Trichoderma saturnisporum* DI-IA from the marine sponge *Dictyonella incisa*	[5]
	Marine-derived *Trichoderma reesei* 4670 from a sponge	[8]
	Marine derived *Trichoderma reesei* from an unidentified sponge	[9]
Trichobisvertinol A (**46**)	Marine-derived *Trichoderma reesei* 4670 from a sponge	[8]
Trichobisvertinol B (**47**)	Marine-derived *Trichoderma reesei* 4670 from a sponge	[8]
Trichobisvertinol C (**48**)	Marine-derived *Trichoderma reesei* 4670 from a sponge	[8]
Trichobisvertinol D = Ustisorbicillinol A (**49**)	Marine-derived *Trichoderma reesei* 4670 from a sponge	[8]
	Rice false smut pathogen *Ustilaginoidea virens*	[6]
12-*epi*-Trichobisvertinol D = Ustisorbicillinol B (**50**)	Marine-derived *Trichoderma reesei* 4670 from a sponge	[8]
	Rice false smut pathogen *Ustilaginoidea virens*	[6]
Trichodermolide B (**51**)	Marine derived *Trichoderma reesei* from a sponge	[9]
13-Hydroxy-trichodermolide (**52**)	Marine derived *Trichoderma reesei* from a sponge	[9]
24-Hydroxy-trichodimerol (**53**)	Marine derived *Trichoderma reesei* from a sponge	[9]
15-Hydroxy-bisvertinol (**54**)	Marine derived *Trichoderma reesei* from a sponge	[9]
13-Hydroxy-dihydrotrichodermolide (**55**)	*Penicillium chrysogernum* 581F1 from the marine sponge *Theonella swinhoei*	[15]
Ustilobisorbicillinol A (**56**)	Rice false smut pathogen *Ustilaginoidea virens*	[11]
Ustisorbicillinol C (**57**)	Rice false smut pathogen *Ustilaginoidea virens*	[6]
Ustisorbicillinol D (**548**)	Rice false smut pathogen *Ustilaginoidea virens*	[6]
Ustisorbicillinol E (**59**)	Rice false smut pathogen *Ustilaginoidea virens*	[6]

Table S3. Occurrence of the hybrid sorbicillinoids (**61-73**) in fungi.

Sorbicillinoid	Fungus and its Origin	Ref.
10-Methylsorbiterrin A (**61**)	*Penicillium* sp. SCSIO06871 from the deep-sea sediment	[2]
Dihydrotrichodermolidic acid (**62**)	*Penicillium* sp. SCSIO06871 from the deep-sea sediment	[2]
Saturnispol C (**63**)	*Trichoderma saturnisporum* DI-IA from the marine sponge *Dictyonella incisa*	[5]
Saturnispol D (**64**)	*Trichoderma saturnisporum* DI-IA from the marine sponge *Dictyonella incisa*	[5]
Spirosorbicillinol D (**65**)	*Trichoderma longibrachiatum* SFC100166 isolated from the foreshore soil	[3]
Sorbicatechol C (**66**)	*Penicillium allii-sativi* from deep-sea	[16]
Sorbicatechol D (**67**)	*Penicillium allii-sativi* from deep-sea	[16]
Sorbicillfuran A (**68**)	Marine-derived fungus *Penicillium citrinum* SCSIO41402	[17]
Sorbicillfuran B (**69**)	Marine-derived fungus *Penicillium citrinum* SCSIO41402	[17]
Sorbicillasin A (**70**)	Deep-sea-derived fungus *Phialocephala* sp. FL30r	[7]
Sorbicillasin B (**71**)	Deep-sea-derived fungus *Phialocephala* sp. FL30r	[7]
Tanshisorbicin (**72**)	Addition of tanshinone IIA in the fermentation broth of *Hypocrea* sp.	[18]
Trichosorbicillin A (**73**)	Marine-derived *Trichoderma reesei* 4670 from a sponge	[8]

Table S4. Cytotoxic activity of the screened sorbicillinoids from fungi.

Sorbicillinoid	Cytotoxic Activity	Ref.
Sorbicillin (**1**)	Cytotoxicity against the human hepatocellular carcinoma cells	[19]
	Antiproliferative activity on HT-29 cells	[16]
24-Hydroxy-trichodimerol (**53**)	Cytotoxic activities on human tumor cells	[16]
Ustilobisorbicillinol A (**56**)	Induced cell cycle arrest, and promoted apoptosis	[11]
Sorbicatechol D (**67**)	Antiproliferative activity on HT-29 cells	[16]
Sorbicillfuran B (**69**)	Weak cytotoxicity against HL-60 cells	[17]
Trichodimerol (**74**)	Moderate cytotoxic activities on human carcinoma cells	[6]
Demethyltrichodimerol (**75**)	Moderate cytotoxic activities on human carcinoma cells	[6]
Dihydrotrichodimer ether A (**76**)	Moderate cytotoxic activities on human carcinoma cells	[6]
Bisvertinolone (**77**)	Moderate cytotoxic activities on human carcinoma cells	[6]

Table S5. Antibacterial activity of the screened sorbicillinoids from fungi.

Sorbicillinoid	Antibacterial Activity	Ref.
Saturnispol F (**9**)	Antibacterial activity on *A. aureus*, VRE, *P. aeruginosa* and *K. pneumonica*	[5]
Saturnispol H (**11**)	Antibacterial activity on VRE and *B. subtilis*	[5]
Ustisorbicillinol B = 12-*epi*-Trichobisvertinol D (**50**)	Antibacterial activity against human/plant pathogenic bacteria	[6]
Tanshisorbicin (**72**)	Antibacterial activity	[18]
Demethyltrichodimerol (**75**)	Antibacterial activity against human/plant pathogenic bacteria	[6]
Dihydrotrichodimer ether A (**76**)	Antibacterial activity against human/plant pathogenic bacteria	[6]
Bisvertinolone (**77**)	Antibacterial activity on *Staphylocossus aureus*	[20]
	Antibacterial activity on *Escherichia coli* and *Pseudomonas lachrymans*	[21]
	Antibacterial activity against human/plant pathogenic bacteria	[6]
Sohirnone A (**78**)	Antibacterial activity	[2]
Dihydrodemethylsorbicillin (**79**)	Antibacterial activity	[2]
Bislongiquinolide (**80**)	Antibacterial activity on *Escherichia coli* and *Pseudomonas lachrymans*	[21]
Oxosorbicillinol (**81**)	Antibacterial activity against human/plant pathogenic bacteria	[6]

Table S6. Antifungal activity of the screened sorbicillinoids from fungi.

Sorbicillinoid	Antifungal Activity	Ref.
Sorbicillin (**1**)	Antifungal activity on *Candida albicans*	[19]
Demethyltrichodimerol (**75**)	Inhibition against the spore germination of rice blast pathogen *Magnaporthe oryzae*	[6]
Bisvertinolone (**77**)	Inhibition against the spore germination of rice blast pathogen *Magnaporthe oryzae*	[6]
	Antifungal activity on phytopathogenic fungi *Cladosporium coccodes*, *Magnaporthe oyrzae* and *Phytopathora infestans*	[3]
Oxosorbicillinol (**81**)	Inhibition against the spore germination of rice blast pathogen *Magnaporthe oryzae*	[6]
	Antifungal activity on phytopathogenic fungi *Cladosporium coccodes*, *Magnaporthe oyrzae* and *Phytopathora infestans*	[3]
Bisorbicillinol (**82**)	Antifungal activity on phytopathogenic fungi *Cladosporium coccodes*, *Magnaporthe oyrzae* and *Phytopathora infestans*	[3]
Epoxysorbicillinol (**83**)	Antifungal activity on phytopathogenic fungi *Cladosporium coccodes*, *Magnaporthe oyrzae* and *Phytopathora infestans*	[3]

Table S7. Anti-inflammatory activity of the screened sorbicillinoids from fungi.

Sorbicillinoid	Anti-Inflammatory Activity	Ref.
2′,3′-Dihydrosorbicillin = Dihydrosorbicillin (**2**)	Inhibition of NO production in RAW264.7 cells activated by LPS	[8]
Trichosorbicillin B (**18**)	Inhibition of NO production in RAW264.7 cells activated by LPS	[8]
Trichosorbicillin C (**19**)	Inhibition of NO production in RAW264.7 cells activated by LPS	[8]
12-Hydroxysorbicillin (**21**)	Inhibition of NO production in RAW264.7 cells activated by LPS	[8]
8,9-Dihydro-12-hydroxysorbicillin (**22**)	Inhibition of NO production in RAW264.7 cells activated by LPS	[8]
Trichosorbicillin E (**23**)	Inhibition of NO production in RAW264.7 cells activated by LPS	[8]
Isotrichosorbicillin E (**24**)	Inhibition of NO production in RAW264.7 cells activated by LPS	[8]
Trichosorbicillin F (**25**)	Inhibition of NO production in RAW264.7 cells activated by LPS	[8]
Trichosorbicilin I (**29**)	Inhibition of NO production in RAW264.7 cells activated by LPS	[8]
Epitetrahydrotrichodimer ether (**39**)	*Penicillium* sp. DM815 form the rhizosphere soil of mangrove *Hibiscus tiliaceus*	[4]
24-Hydroxybisvertinol (**45**)	Inhibition of NO production in RAW264.7 cells activated by LPS	[8]
Trichobisvertinol A (**46**)	Inhibition of NO production in RAW264.7 cells activated by LPS	[8]
Trichobisvertinol B (**47**)	Inhibition of NO production in RAW264.7 cells activated by LPS	[8]
Trichobisvertinol C (**48**)	Inhibition of NO production in RAW264.7 cells activated by LPS	[8]
Trichobisvertinol D (**49**)	Inhibition of NO production in RAW264.7 cells activated by LPS	[8]
12-*epi*-Trichobisvertinol D (**50**)	Inhibition of NO production in RAW264.7 cells activated by LPS	[8]
Trichodimerol (**74**)	Neural anti-inflammatory activity	[22]
Sohirnone A (**78**)	Inhibition of NO production in RAW264.7 cells activated by LPS	[8]
Sorrentanone (**84**)	Neural anti-inflammatory activity	[22]
Bisvertinol (**85**)	Inhibition of NO production in RAW264.7 cells activated by LPS	[8]
(2*E*,4*E*)-1-(2,6-Dihydroxy-3,5-dimethylphenyl)hexa-2,4-dien-1-one (**86**)	Inhibition of NO production in RAW264.7 cells activated by LPS	[8]
Trichodermanone C (**87**)	Inhibitory effect on nitrite production in LPS-stimulated marophages	[23]
Tetrahydrotrichodimerol (**88**)	*Penicillium* sp. DM815 form the rhizosphere soil of mangrove *Hibiscus tiliaceus*	[4]

Table S8. Phytotoxic activity of the screened sorbicillinoids from fungi.

Sorbicillinoid	Phytotoxic Activity	Ref.
(-)-(*S*)-Dihydrovertinolide (**38**)	Phytotoxic activity against lettuce seedlings	[13]
Trichodimerol (**74**)	Inhibition against the radicle and germ elongation of rice and lettuce seedlings	[6]
Demethyltrichodimerol (**75**)	Inhibition against the radicle and germ elongation of rice and lettuce seedlings	[6]
Bisvertinolone (**77**)	Inhibition against the radicle and germ elongation of rice and lettuce seedlings	[6]
Bislongiquinolide (**80**)	Inhibition against the radicle and germ elongation of rice and lettuce seedlings	[6]

Table S9. α–Glucosidase inhibitory activity of the screened sorbicillinoids from fungi.

Sorbicillinoid	Biological Activity	Ref.
2′,3′-Dihydrosorbicillin = Dihydrosorbicillin (**2**)	α-Glucosidase inhibitory activity	[24]
5-Hydroxy-dihydrodemethylsorbicillin (**15**)	α-Glucosidase inhibitory activity	[2]
Bisorbicillpyrone A (**41**)	α-Glucosidase inhibitory activity	[2]
Dihydrodemethylsorbicillin (**79**)	α-Glucosidase inhibitory activity	[2]
Tetrahydrotrichodimerol (**88**)	α-Glucosidase inhibitory activity	[2]
Tetrahydrobisvertinolone (**89**)	α-Glucosidase inhibitory activity	[2]
10,11-Dihydrobisvertinolone (**90**)	α-Glucosidase inhibitory activity	[2]

Table S10. Other biological activities of the screened sorbicillinoids from fungi.

Sorbicillinoid	Biological Activity	Ref.
Sorbicillin (**1**)	Inhibitory activity on acetylcholinesterase	[24]
	Neuroprotective and neuritogenic activity	[25]
2′,3′-Dihydrosorbicillin = Dihydrosorbicillin (**2**)	Inhibitory activity on acetylcholinesterase	[24]
	Inhibitory activity on protein tyrosine phosphatase 1B	[26]
Scipyrone K (**14**)	Antioxidant activity	[7]
Trichoreesin A (**32**)	Antimicroalgal activity against the marine algae	[10]
13-Hydroxy-dihydrotrichodermolide (**55**)	Inhibitory activities on glucagon-like peptide-1 and. eukaryotic elongation factor-2 kinase	[15]
10,11,27,28-Tetrahydrotrisorbicillinone C (**60**)	Inhibitory activities on glucagon-like peptide-1 and. eukaryotic elongation factor-2 kinase	[15]
Sohirnone A (**78**)	Inhibitory activity on protein tyrosine phosphatase 1B	[26]
Bisorbicillinol (**82**)	Anti-allergic activity	[27]
Sorrentanone (**84**)	Anti-HIV activity	[22]
Tetrahydrobisvertinolone (**89**)	Weak AChE inhibitory activity	[2]
Tetrahydrotrichodimer ether (**91**)	Weak AChE inhibitory activity	[2]

Supplementary References

1. Jiang, C.-S.; Zhou, Z.-F.; Yang, X.-H.; Lan, L.-F.; Gu, Y.-C.; Ye, B.-P.; Guo, Y.-W. Antibacterial sorbicillin and diketopiperazines from the endogenous fungus *Penicillium* sp. GD6 associated Chinese mangrove *Bruguiera Gymnorrhiza*. *Chin. J. Nat. Med.* **2018**, *16*, 358–365.
2. Pang, X.; Zhou, X.; Lin, X.; Yang, B.; Tian, X.; Wang, J.; Xu, S.; Liu, Y. Structurally various sorbicillinoids from the deep-sea sediment derived fungus *Penicillium* sp. SCSIO06871. *Bioorg. Chem.* **2021**, *107*, 104600.
3. Ngo, M.T.; Nguyen, M.V.; Han, J.W.; Park, M.S.; Kim, H.; Choi, G.J. *In vitro* and *in vivo* antifungal activity of sorbicillinoids produced by *Trichoderma Longibrachiatum*. *J. Fungi* **2021**, *7*, 428.
4. Ding, W.; Wang, F.; Li, Q.; Xue, Y.; Dong, Z.; Tian, D.; Chen, M.; Zhang, Y.; Hong, K.; Tang, J. Isolation and characterization of anti-inflammatory sorbicillinoids from the mangrove-derived fungus *Penicillium* sp. DM815. *Chem. Biodivers.* **2021**, *18*, e2100229.
5. Meng, J.; Cheng, W.; Heydari, H.; Wang, B.; Zhu, K.; Konuklugil, B.; Lin, W. Sorbicillinoid-based metabolites from a sponge-derived fungus *Trichoderma saturnisporum*. *Mar. Drugs* **2018**, *16*, 226.

6. Meng, J.; Gu, G.; Dang, P.; Zhang, X.; Wang, W.; Dai, J.; Liu, Y.; Lai, D.; Zhou, L. Sorbicillinoids from the fungus *Ustilaginoidea virens* and their phytotoxic, cytotoxic, and antimicrobial activities. *Front. Chem*. **2019**, *7*, 435.
7. Zhang, Z.; He, Q.; Che, Q.; Zhang, G.; Zhu, T.; Gu, Q.; Li, D. Sorbicillasins A–B and scirpyrone K from a deep-sea-derived fungus, *Phialocephala* sp. FL30r. *Mar. Drugs* **2018**, *16*, 245.
8. Zhang, P.; Deng, Y.; Lin, X.; Chen, B.; Li, J.; Liu, H.; Chen, S.; Liu, L. Anti-inflammatory mono- and dimeric sorbicillinoids from the marine-derived fungus *Trichoderma reesei* 4670. *J. Nat. Prod*. **2019**, *82*, 947–957.
9. Rehman, S.U.; Yang, L.-J.; Zhang, Y.-H.; Wu, J.-S.; Shi, T.; Halder, W.; Shao, C.-L.; Wang, C.-Y. Sorbicillinoid derivatives from sponge-derived fungus *Trichoderma reesei* (HN-2016-018). *Front. Microbiol*. **2020**, *11*, 1334.
10. Ma, X.-Y.; Shi, Z.-Z.; Ji, N.-Y. Sorbicillinoids from the alga-epiphytic fungus *Trichoderma reesei* Z56-8. *Nat. Prod. Res*. **2021**. https://doi.org/10.1080/14786419.2021.1980793.
11. Lai, D.; Meng, J.; Zhang, X.; Xu, D.; Dai, J.; Zhou, L. Ustilobisorbicillinol A, a cytotoxic sorbyl-containing aromatic polyketide from *Ustilagninoidea virens*. *Org. Lett*. **2019**, *21*, 1311–1314.
12. McMullin, D.R.; Renaud, J.B.; Barasubiye, T.; Sumarah, M.W.; Miller, J.D. Metabolites of *Trichoderma* species isolated from damp building materials. *Can. J. Microbiol*. **2017**, *63*, 621–632.
13. Supratman, U.; Suzuki, T.; Nakamura, T.; Yokoyama, Y.; Harneti, D.; Maharani, R.; Salam, S.; Abdullah, F.; Koseki, T.; Shiono, Y. New metabolites produced by endophyte *Clonostachys rosea* B5-2. *Nat. Prod. Res*. **2021**, *35*, 1525–1351.
14. Yu, J.; Han, H.; Zhang, X.; Ma, C.; Sun, C.; Che, Q.; Gu, Q.; Zhu, T.; Zhang, G.; Li, D. Discovery of two new sorbicillinoids by overexpression of the global regulator LaeA in a marine-derived fungus *Penicillium dipodomyis* YJ-11. *Mar. Drugs* **2019**, *17*, 446.
15. Cao, M.-J.; Zhu, T.; Liu, J.-T.; Ouyang, L.; Yang, F.; Lin, H.-W. New sorbicillinoid derivatives with GLP-1R and eEF2K affinities from a sponge-derived fungus *Penicillium Chrysogenum* 581F1. *Nat. Prod. Res*. **2020**, *34*, 2880–2886.
16. Xie, C.-L.; Zhang, D.; Lin, T.; He, Z.-H.; Yan, Q.-X.; Cai, Q.; Zhang, X.-K.; Yang, X.-W.; Chen, H.-F. Antiproliferative sorbicillinoids from the deep-sea-derived *Penicillium allii-sativi*. *Front. Microbiol*. **2021**, *11*, 636948.
17. Wang, J.; Li, K.; Luo, X.; Wu, Z.; Gu, T.; Liao, S.; Lin, X.; Yang, B.; Liu, Y.; Fang, W.; et al. Sorbicillfurans A and B, two novel sorbicillinoid adducts from the fungus *Penicillium citrinum* SCSIO41402. *Org. Biomol. Chem*. **2019**, *17*, 8721.
18. He, W.; Liu, M.; Li, X.; Zhang, X.; Abdel-Mageed, W.M.; Li, L.; Wang, W.; Zhang, J.; Han, J.; Dai, H.; et al. Fungal biotransformation of tanshinone results in [4+2] cycloaddition with sorbicillinol: Evidence for enzyme catalysis and increased antibacterial activity. *Appl. Microbiol Biotechnol*. **2016**, *100*, 8349–8357.
19. Lin, S.; Wu, Y.-Z.; Chen, K.-Y.; Ye, J.; Yang, X.-W.; Zhang, W.-D. Polyketides from the fungus *Penicillium decumbens*. *J. Asian Nat. Prod. Res*. **2018**, *20*, 445–450.
20. Corral, P.; Esposito, F.P.; Tedesco, P.; Falco, A.; Tortorella, E.; Tartaglione, L.; Festa, C.; D'Auria, M.V.; Gnavi, G.; Varese, G.C.; et al. Identification of a sorbicillinoid-producing *Aspergillus* strain with antimicrobial activity against *Staphylococcus aureus*: A new polyextremophilic marine fungus from Barents Sea. *Mar. Biotechnol*. **2018**, *20*, 502–511.
21. Meng, J.; Wang, B.; Cheng, W. Study on the secondary metabolites of *Thichoderma sturnisporum*. *Chin. J. Mar. Drugs* **2017**, *36*, 27–31.
22. Zhao, J.-L.; Zhang, M.; Liu, J.-M.; Tan, Z.; Chen, R.-D.; Xie, K.-B.; Dai, J.-G. Bioactive steroids and sorbicillinoids isolated from the endophytic fungus *Trichoderma* sp. Xy24. J. *Asian Nat. Prod. Res*. **2017**, *19*, 1028–1035.
23. Marra, R.; Nicoletti, R.; Pagano, E.; DellaGreca, M.; Salvatore, M.M.; Borrelli, F.; Lombardi, N.; Vinale, F.; Woo, S.L.; Andolfi, A. Inhibitory effect of trichodermanone C, a sorbicillinoid produced by *Trichoderma citrinoviride* associated to the green alga *Cladophora* sp., on nitrite production in LPS-stimulated macrophages. *Nat. Prod. Res*. **2019**, *33*, 3389–3397.
24. Deng, J.; Dai, H.; Wang, Y.; Chen, H.; Tan, Z.; Mei, W. Isolation and identification of the fungus *Aspergillus* sp. HNWSW-20 from Chinese agarwood and its secondary metabolites. *Chin. J. Trop. Crops* **2018**, *39*, 1618–1624.

25. Tilki, E.K.; Ozturk, S.E.; Ozarda, M.G.; Canturk, Z.; Dikmen, M. Investigation of the neuroprotective and neuritogenic effects of halotolerant *Penicillium flavigenum*-derived sorbicillin-like compounds on PC-12 Adh cells. *Cytotechnology* **2021**, *73*, 801–813.
26. Han, X.; Tang, X.; Qiao, D.; Li, P.; Li, G. Studies on bioactive components of a marine-derived fungus *Penicillium chrysogenum*. *Chin. J. Mar. Drugs* **2019**, *38*, 77–81.
27. Sugaya, K.; Terajima, T.; Takahashi, A.; Onose, J.; Abe, N. Bisorbicillinol inhibits Lyn tyrosine kinase for allergic response on RBL-2H3 cells. *Bioorg. Med. Chem. Lett*. **2019**, *29*, 832–835.
